# Encephaloid Mass Penetrating the Right Renal Vein and the Vena Cava

**Published:** 1867-09

**Authors:** 


### ENCEPHALOID MASS PENETRATING THE RIGHT RENAL VEIN AND THE VENA CAVA.

Dr. Bogu exhibited a very remarkable specimen of cancerous growth, which he had removed from a patient in the Cook County Hospital.

The patient, aged 35 years, male, entered the hospital in May, having suffered about two months from pain in the left side of the abdomen and chest, hæmoptysis, coldness of the extremities, night sweats, and loss of appetite. The erect posture produced dizziness and blindness; the horizontal posture produced tinnitus. There was crepitation in each lung, nausea, constipation, and little urine; the right leg was paralized, enormously œdematous, as far as the middle of the thigh, and covered with a large number of black patches. There were many dark colored nodules, varying in size from that of a pea to that of a pigeon's egg, on the scalp, back, and along the course of the lymphatics. There was also an abdominal tumor above the umbilicus, which had escaped the notice of the patient. At last there was pain in the bladder, with retention of the urine, and œdema of the left foot. Death occurred a few days after the patient's entrance into the hospital.

The autopsy revealed a large encephaloid mass, situated over the lumbar vertebra, which were found diseased into the very cavity of the spinal canal. The mass involved the vena cava and right renal vein, penetrating their walls, and nearly filling their cavities with a soft fungus mass. There were deposits of cancerous structure in the lungs, liver, and abdominal glands.

